# Application of purse string suture pancreaticojejunostomy for undilated pancreatic duct in total laparoscopic pancreaticoduodenectomy

**DOI:** 10.1186/s12893-022-01640-w

**Published:** 2022-05-19

**Authors:** Haihua Zhou, Shian Yu, Xiaokang Wu, Xuemin Li

**Affiliations:** Department of Hepatobiliary Surgery, The Affiliated Jinhua Hospital of Zhejiang University School of Medicine, Jinhua, Zhejiang China

**Keywords:** Laparoscopy, Pancreaticoduodenectomy, Pancreaticojejunostomy, Pancreatic fistula, Undilated pancreatic duct

## Abstract

**Background:**

To investigate the feasibility of purse string suture pancreaticojejunostomy in complete laparoscopic pancreaticoduodenectomy for patients with an undilated pancreatic duct.

**Methods:**

We retrospectively reviewed a database of 113 patients with undilated pancreatic ducts who had undergone laparoscopic pancreaticoduodenectomy (LPD) with purse string suture pancreaticojejunostomy to analyze the perioperative outcomes.

**Results:**

One hundred thirteen patients underwent successful LPD. The surgery time was 353 ± 41 min, the time required for pancreaticojejunostomy was 27 ± 5 min, and the hospital stay after surgery was 16 ± 8 days. Fifteen patients suffered postoperative complications, including twelve patients with pancreatic fistula, one with bile leakage, one with gastroparesis (complicated with abdominal infection), and one with abdominal bleeding. No perioperative death occurred.

**Conclusions:**

Purse string suture pancreaticojejunostomy is safe and feasible for patients with an undilated pancreatic duct.

**Supplementary Information:**

The online version contains supplementary material available at 10.1186/s12893-022-01640-w.

## Background

The treatment and anastomosis of the pancreatic stump are the most complicated aspects of pancreaticoduodenectomy (PD), especially for patients with an undilated pancreatic duct, and are key factors affecting pancreatic fistula. Pancreatic fistula is a common and serious complication of pancreaticoduodenectomy and the most important factor affecting the prognosis. Pancreaticojejunal anastomosis in patients with an undilated pancreatic duct has certain technical difficulties. Although the technology is developing, the incidence of postoperative pancreatic fistula (POPF) is still 10–25% [1–5]. Although there are various types of pancreaticojejunostomy [PJ] [6], few reports have focused on anastomosis in patients with an undilated main pancreatic duct, especially for laparoscopic pancreaticoduodenectomy, and there is no evidence of the kind of anastomosis that best avoids the occurrence of pancreatic leakage, especially in patients with an undilated pancreatic duct. Our team has continuously explored methods of laparoscopic pancreaticojejunostomy, and has adopted a new type of pancreaticoduodenectomy in patients with an undilated pancreatic duct, which we call ‘purse string suture pancreaticojejunostomy’. We have summarized the data on patients who underwent pancreaticoduodenectomy between January 2013 and December 2021, and analyzed the perioperative conditions of the patients with an undilated pancreatic duct to determine the clinical effect of purse string suture pancreaticojejunostomy in total laparoscopic pancreaticoduodenectomy. Our purpose was to demonstrate our method and to offer further anastomosis options for patients with an undilated main pancreatic duct.

## Methods

**Fig. 1:**
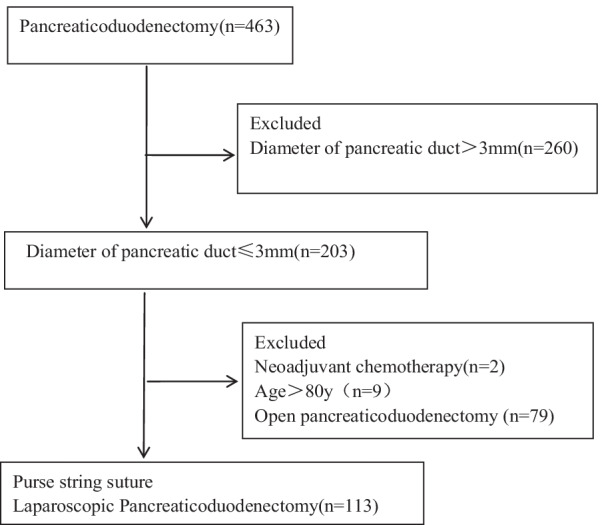
Flowchart of patient allocation

From January 2013 to December 2021,463 consecutive patients underwent pancreatoduodenectomy at the Department of Hepatobiliary Surgery, the Affiliated Jinhua Hospital of Zhejiang University School of Medicine. 260 patients with the diameter of pancreatic duct > 3 mm were excluded. 203 with the diameter of pancreatic duct ≤ 3 mm were enrolled in this study. Among the 203 patients, 9 patients older than 80y, 2 patients received neoadjuvant chemotherapy and 79 patients received open pancreaticduodenectomy were excluded. Patient allocation in this study is summarized in Fig. 1. In total 113 patients were analyzed, including 64 females and 49 males aged 18–80 years. All patients was diagnosed with periampullary tumors with computed tomography (CT), magnetic resonance imaging (MRI), endoscopic ultrasonography, or positron emission tomography–CT before surgery. The preoperative data, including age, sex, body mass index, albumin, and total bilirubin, were analyzed systematically to evaluate each patient’s general condition and ability to tolerate the procedure. Ten patients were treated with biliary drainage before surgery (Table 1). All the patients were deemed able to tolerate the laparoscope surgery. The pathological diagnoses were clear during or after the operation. Patients with tumor metastasis to other sites were excluded. No tumor involved the abdominal aorta, common hepatic artery, or mesenteric artery. No patient had undergone neoadjuvant chemotherapy before surgery. Three patients were diagnosed with a tumor invading the portal vein side wall (< 180°) before surgery. The diameter of the pancreatic duct was measured during surgery, and all were ≤ 3 mm. The informed consent of the surgery was obtained from all patients or their legal guardians. This study was approved by the Institutional Review Board of the Affiliated Jinhua Hospital, Zhejiang University School of Medicine, Jinhua, China.

**Table 1:** Patients demographics and clinical findings

	N = 113
Age (years)	61 ± 10 (27–80)
Female/male (n)	64/49
Body mass index (kg/m^2^)	22.73 ± 2.34 (17–28)
Preoperative total bilirubin of blood (μmol/L)	98.32 ± 89.67 (8.6–352)
Preoperative blood albumin (g/L)	38.05 ± 4.23 (30–48.7)
Preoperative biliary drainage (n)	
PTCD	4
PTGD	2
ENBD	4

*PTCD* percutaneous transhepatic cholangial drainage, *PTGD* percutaneous transhepatic gallbladder drainage, *ENBD* endoscopic nasobiliary drainage

### Surgical procedure

#### Posture and trocar positions

The patients lay in a supine position with legs slightly higher. A trocar with a diameter of 1.0 cm was inserted into the lower edge of the umbilicus as the laparoscope port. Another four trocars were inserted: one 12 mm trocar on the right midclavicular line for the right hand of the first assistant; one 12 mm trocar on the left midclavicular line for the right hand of the operator; and two additional 5 mm trocars (one on the left flank for the left hand of the operator and the other on the right midclavicular line for the left hand of the first assistant). Thus, the trocars had a V-shaped distribution. The abdominal cavity was filled with carbon dioxide gas, at a pressure of 12–15 mmHg. The operator and the first assistant stood on the left and right side of the patient, respectively, with the laparoscope holder between the patient’s legs. The digestive tract was reconstructed with the Child method.

#### Specimen resection and reconstruction of digestive tract

We used an ultrasonic knife to open the gastrocolic ligament to expose the pancreas, and dissected the lower edge of the pancreas to establish a posterior pancreatic tunnel in front of the superior mesenteric vein and portal vein. We then dissected the upper edge of the pancreas to expose the splenic artery, common hepatic artery, and gastroduodenal artery, and simultaneously dissected all the lymph nodes in this area. After the resectability of the tumor was assessed, the stomach was resected with the endovascular gastrointestinal anastomosis stapler (Endo GIA), and then the pancreas was cut off with electrocautery, making it easy to identify the pancreatic duct. The main pancreatic duct was found and the stent was placed first. We measured the diameter of the pancreatic duct of the pancreatic stump with suture in abdomen at the time of dissection of the pancreatic duct, and then measured the suture with a high-precision caliper outside the abdomen. The uncinate process was cut along the right margin of the superior mesenteric artery in the end. In three patients in whom the tumor had invaded the side wall of the portal vein, a wedge resection of the side wall was included during the operation, and the vein was repaired with a continuous suture of 4-0 Prolene. If the diameter of the bile duct was less than 1.0 cm, we used an intermittent suture of polydloxanone suture (PDS) to reconstruct the biliary-enteric anastomosis, but otherwise used a continuous suture.

#### Pancreaticojejunostomy

**Fig. 2:**
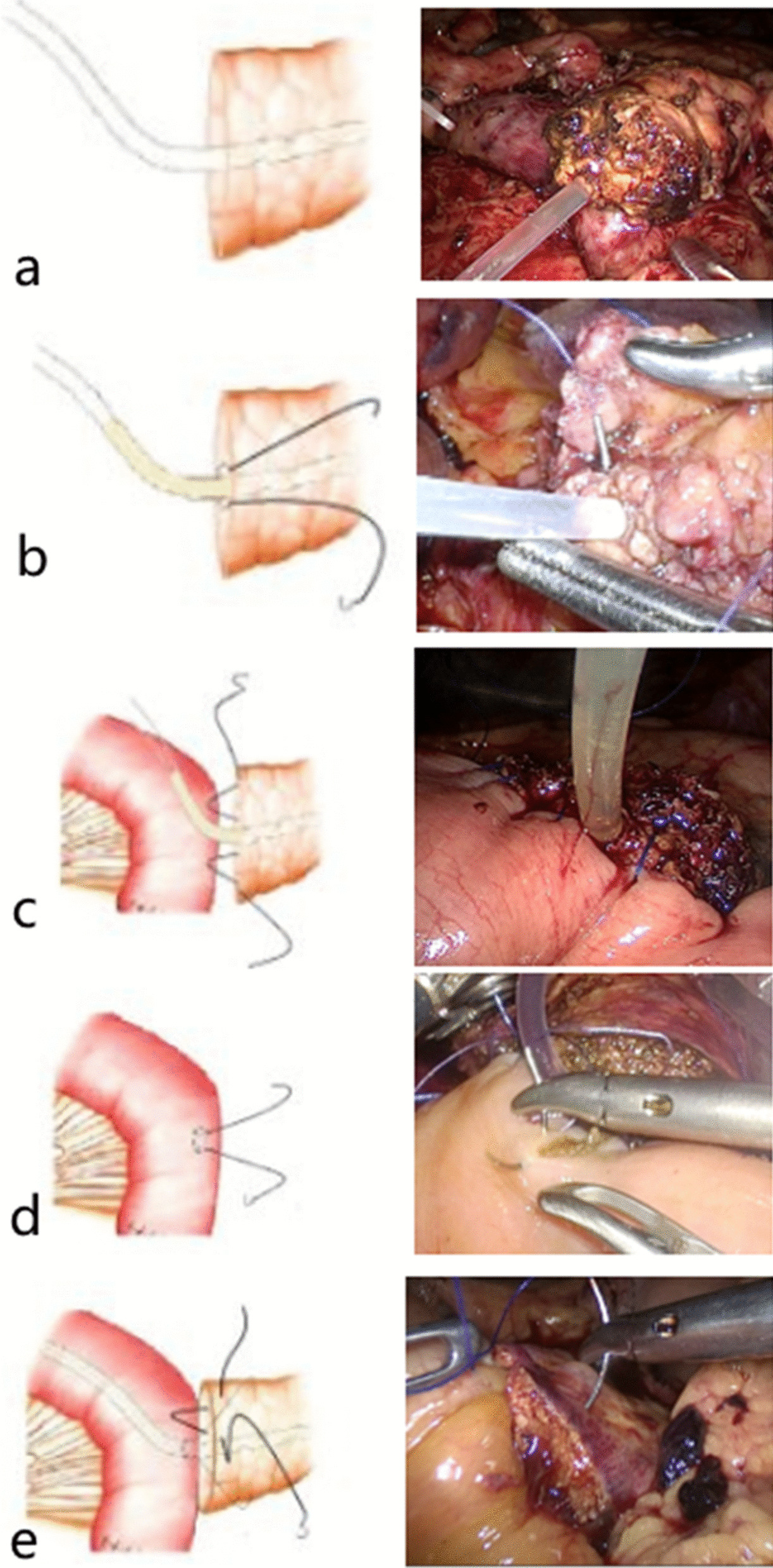
**a** An appropriate length of stenting tube was inserted into the main pancreatic duct. **b** The pancreatic stump was sutured with a 4-0 absorbable suture around the pancreatic duct, about 3 mm away from it, into a purse suture and the stent in the pancreatic duct was fixed with knot. **c** The posterior side of the pancreatic stump and the jejunal sarcomuscular layer were sutured with 3-0 Prolene suture. **d** Running pouch suture throughout the full thickness of intestinal wall surrounded the stent, about 3 mm from the jejunal incision, and tying the knot tightly. **e** Suture the anterior side of pancreatic stump and jejunum sarcomuscular layer with prolene thread

All the patients were treated with purse string suture pancreaticojejunostomy (Additional file 1) and the anastomosis was performed at an appropriate position about 5 cm from the blind end of the jejunal loop. An appropriate length of stenting tube (silica gel tube) was inserted into the main pancreatic duct (Fig. 2a). The diameter of the stenting tube was similar to that of the main pancreatic duct, and 3–4 small holes were cut into the lateral wall of the tube to facilitate the drainage of pancreatic fluid. The pancreatic stump was sutured with 4-0 absorbable suture around the pancreatic duct, about 3 mm away from it, into a purse suture (Fig. 2b), then a tight knot was made. The stent in the pancreatic duct was fixed with the knot, which was made as tight as possible to avoid the outflow of succus pancreaticus from the space between the stent and the pancreatic duct. The posterior side of the pancreatic stump and the jejunal sarcomuscular layer were sutured with 3-0 Prolene suture continuously (Fig. 2c), and the exit point of the needle in the pancreas was about 1 cm from the pancreatic stump to avoid cutting or tearing the pancreas. Five to eight continuous stitches were used to complete the reconstruction of the posterior wall of the pancreaticojejunal anastomosis. In this process, we maintained proper needle spacing, tightened the suture, and ensured that the posterior edge of the pancreas and the jejunal sarcomuscular layer were sutured together. We then used an electrotome to cut a small incision, similar to the pancreatic duct, in the contralateral mesangium bowel. We inserted the distal part of the main pancreatic duct stenting tube about 5 cm into the lumen, and performed a pouch suture throughout the full thickness of the intestinal wall surrounding the stent, about 3 mm from the jejunal incision (Fig. 2d), and tied the knot tightly, thus ensuring that the jejunum incision fitted the pancreatic duct as far as possible to avoid the outflow of intestinal fluid. We continued to suture the anterior side of the pancreatic stump and the jejunum sarcomuscular layer with Prolene suture to complete the anterior wall of the pancreaticojejunal anastomosis (Fig. 2e). The needle exit point at the pancreatic stump in the anterior layer was lower than the entry point at the pancreatic stump in the posterior wall so that there was a partial overlap plane between the anterior and posterior layers, to ensure that all the pancreatic parenchyma was penetrated by the suture, and at the same time, the surface of the pancreatic stump was completely fitted to the jejunum.

Two drainage tube were placed posterior and anterior to the pancreaticointestinal anastomosis.

### Statistical analysis

Data were collected for all patients (Additional file 2), including patient demographics, pathological findings, perioperative clinical information, and complications (Table 2). Data are expressed as medians and ranges. The patient characteristics and perioperative and postoperative factors were analyzed with Student’s *t* test. All statistical analyses were performed with the SPSS 25.0 software program (SPSS Inc., Chicago, IL, USA).

**Table 2:** Patients demographics and clinical findings

	N = 113
Operative time (min)	353 ± 41 (280–470)
Duration of PJ (min)	27 ± 5 (20–40)
Diameter of Wirsung duct (n)	
≤ 3 mm	113
Texture pancreas (hard/soft) (n)	64/49
Blood loss (mL)	276 ± 213 (100–1000)
Postoperative hospital stay (days)	16 ± 8 (7–70)
Histologic diagnosis (n)	
Pancreatice adenocarcinoma	26
Duodenal adenocarcinoma	28
Ampullary cancer	29
Bile duct cancer	27
Chronic pancreatitis	1
IPMN	1
Neuroendocrine carcinoma	1
Amylase level on drain fluid (IU/mL) (normal range 20–130)	
POD 1	42.34 ± 33.99
POD 3	118.06 ± 202.13
POD 5	146.38 ± 301.65
Perianastomotic drain output (mL)	
POD 3	131 ± 79
POD 5	141 ± 74
Days of drain tube placement (days)	13 ± 9 (7–63)
POPF, n (%)	
Biochemical leakage (BL)	10 (8.85%)
B grade	11 (9.73%)
C grade	1 (0.88%)
Abdominal bleeding (n)	1
Gastroparesis (n)	1
Bile leakage (n)	1
Re-operation (n)	2
Mortality (n)	0

### Postoperative management

Prophylactic octreotide or somatostatin was not routinely used. An oral diet was resumed on postoperative day (POD) 3. Serum amylase and the amylase concentration in the fluid collected from the peripancreatic drain were measured on PODs 1, 3, and 5. The drainage tube was removed when the drainage fluid was clear, the drainage volume was < 100 mL, and the drainage fluid amylase was within the normal range.

## Results

All 113 patients were treated with complete laparoscopy. The diameter of the pancreatic duct was ≤ 3 mm when measured during the operation. The operation time was 280–470 min, with an average of 353 ± 41 min. The reconstruction time of the pancreaticojejunostomy was 20–40 min, with an average of 27 ± 5 min. Intraoperative blood loss was 100–1000 mL. In three cases, the lateral portal vein was resected, and was reconstructed with a continuous suture of 4-0 Prolene suture. The postoperative hospital stay was 7–70 days, with an average of 16 ± 8 days. Postoperative complications occurred in 15 patients, including grade C pancreatic fistula in one patient, grade B pancreatic fistula in 11 patients, abdominal hemorrhage in one patient, bile leakage in one patient, and gastroparesis in one patient. Pancreatic fistula was diagnosed according to the definition and grading criteria of the International Study Group of Pancreatic Surgery (ISGPS, 2016 edition) [7]. Ten patients were diagnosed with biochemical leakage. One patient suffered grade C postoperative pancreatic fistula (POPF) complicated with abdominal infection and bloody drainage fluid was collected in the abdominal tube 12 days after surgery. When the patient underwent exploratory laparotomy during the operation, no clear bleeding point was found, but there was pyogenic fluid around the pancreaticointestinal anastomosis, so debridement and drainage were performed. The patient was treated successfully and discharged 32 days after surgery. Eight patients with grade B POPF were cured with conservative treatment, whereas three other patients with grade B POPF had a poor outcome after conservative treatment, complicated with abdominal infection, and were cured with abdominal puncture and ultrasound-guided drainage. Patients with POPF received abdominal CT examination timely, and all stents inserted in the pancreatic duct were in place. Abdominal hemorrhage accompanied by hematemesis occurred in one patient on the first day after surgery. Digital subtraction angiography (DSA) revealed bleeding in the left gastric artery branch, and interventional embolization was successfully performed to stop the bleeding. One patient with gastroparesis (combined with abdominal infection) was hospitalized for 70 days after surgery, and was cured with conservative treatment. Pancreatitis was not detected in any patients postoperatively. Postoperative pathology showed ampullary carcinoma in 29 patients, duodenal carcinoma in 28 patients, pancreatic carcinoma in 26 patients, common bile duct carcinoma in 27 patients, pancreatic head neuroendocrine carcinoma in one patient, pancreatic intraductal papillary mucinous neoplasm in one patient, and chronic pancreatitis in one patient. Ninety-two patients underwent an abdominal CT scan 3 months after surgery, and we found the stent inserted into the pancreaticointestinal anastomosis had been dislodged in 76 patients (82.61%), whereas the stents of 16 patients were still in the pancreaticointestinal anastomosis.

## Discussion

Risk factors for pancreatic fistula include both patient factors and iatrogenic factors [8–10]. An undilated pancreatic duct is one of the risk factors for pancreatic fistula after pancreaticojejunostomy [11, 12]. Pancreaticojejunostomy is the key factor in the development of pancreatic fistula and affects the outcome of the disease [10, 13].

The methods of pancreaticojejunostomy can be summarized into three main types: invagination pancreaticojejunostomy, duct-to-mucosa pancreaticojejunostomy, and binding pancreaticojejunostomy. Pancreaticojejunostomy is difficult in patients with an undilated pancreatic duct. Duct-to-mucosa pancreaticojejunostomy has become a highly recommended method in recent years. Studies have shown that this method has the advantages of maintaining anastomotic patency and the remaining pancreatic exocrine function. Theoretically, the larger the diameter of the pancreatic duct, the greater the safety of this procedure. However, laparoscopic surgery entails certain technical difficulties, especially for patients with undilated pancreatic duct, and inexact anastomosis may increase the incidence of postoperative pancreatic fistula [14, 15].

The process of invagination and banding are complicated and time-consuming in laparoscopic surgery, and the incidence of anastomotic bleeding is especially high when the pancreatic stump is directly exposed to digestive juices. If the pancreatic stump is too large, it is difficult to insert into the intestinal lumen. Forced anastomosis may lead to pancreatic congestion, edema, or even necrosis, and increase the incidence of pancreatic fistula [16].

Our method, which is used in patients with an undilated main pancreatic duct, involves a purse string suture at the jejunum incision and pancreatic duct, and bridges the pancreatic duct and jejunum with a stenting tube. Therefore, we named this method ‘purse string suture pancreaticojejunostomy’. Our approach simplifies the surgical steps, shortens the operation time, reduces the technical difficulty, and can be easily performed laparoscopically. The rate of POPF (grade B/C) was 9.73% in this study. When we searched the database, we found few articles focusing on patients with an undilated main pancreatic duct. Kawakatsu [17] reported that the POPF (grade B/C) rate was 47.26% (95/201) in patients treated with duct-to-mucosa pancreaticojejunostomy during open pancreaticoduodenectomy, when the main pancreatic ducts was  < 4 mm. Nagakawa [18] reported the incidence of POPF (grade B/C) to be 20% (4/20) and 21% (4/19) in patients with a soft pancreas but with no pancreatic duct dilation treated with conventional Blumgart PJ and Blumgart PJ with LAPRA-TY respectively during laparoscopic pancreaticoduodenectomy, and the mean operation time of pancreaticojejunostomy was 69.7 ± 13.1 min and 56.2 ± 10.5 min respectively, which were both longer than us.

With pancreatic leakage, pancreatic fluid first accumulates in the anastomotic space between the pancreatic stump and the jejunal serosal surface, and then flows into the intraperitoneal space after it has accumulated to a certain extent [19]. The gap between the stenting tube, the pancreatic duct, and the jejunal incision is tensioned with a purse string suture in our method, to avoid the pancreas section being soaked in digestive juice. The supporting stent guarantees the patency of the pancreaticojejunostomy and the exocrine function of the residual pancreas. The healing of the pancreatic duct and jejunal mucosa occurs simultaneously. Theoretically, pancreatitis may be occur if the stent was occluded. Postoperative pancreatitis was not detected in any patients in our study. 3–4 small holes were cut into the lateral wall of the tube to facilitate the drainage of pancreatic fluid, which reduces the possibility of blockage of the stent.

In open PD surgery, many surgeons now use a mattress suture or an intermittent suture between the pancreatic stump and the jejunal seromuscular layer [20]. Regardless of which approach is taken, the purpose is to ensure close adherence of the pancreatic stump and the jejunal sarcomuscular layer. The purse string anastomosis technique involves continuous suturing, which is one of the mature surgical suturing techniques. The scope of its application has been limited in the past by the suture materials available. With the improvement of suture materials, continuous suturing is a quick and simple technique.

Pancreaticoduodenectomies with a duct-to-mucosa pancreaticojejunostomal anastomosis, with or without a stenting tube, did not differ at the long-term follow-up [21]. However, the stenting tube plays an important role in our technique. The patency of the pancreaticojejunostomy is mainly ensured by bridging the pancreatic duct stent during the perioperative period. The stent is similar to the pancreatic duct so the pancreatic fluid is less likely to leak out around the duct. The anterior and posterior edges of the pancreatic section intersect with the jejunal sarcomuscular layer, and the pancreatic section is closely sutured to the intestinal wall to avoid the accumulation of fluid at the anastomosis surface, ensuring the rapid and close adherence of the anastomosis. This may also effectively prevent bleeding from the pancreatic section and any leakage of pancreatic fluid.

## Conclusions

This method simplifies the performance of laparoscopic pancreaticojejunostomy. The method is safe and feasible for laparoscopic pancreaticoduodenectomy in patients with undilated main pancreatic duct. We still use duct-to-mucosa anastomosis for patients with pancreatic duct diameters of > 3 mm. Further research is required to determine whether purse string suture pancreaticojejunostomy is suitable for patients with dilated pancreatic ducts.

## Supplementary Information

**Additional file 1.** Surgery video.

**Additional file 2.** Raw data.

## Data Availability

All data and materials are fully available without restriction. All data generated or analysed during this study are included in this published article.
